# Turner syndrome: skin, liver, eyes, dental and ENT evaluation should be improved

**DOI:** 10.3389/fendo.2023.1190670

**Published:** 2023-07-25

**Authors:** Jenny Lam, Sophie Stoppa-Vaucher, Maria Cristina Antoniou, Thérèse Bouthors, Inge Ruiz, Nicole Sekarski, Tobias Rutz, Sophie Fries, Pierre Alain Binz, Florence Niel Bütschi, Nicolas Vulliemoz, Aneta Gawlik, Nelly Pitteloud, Michael Hauschild, Kanetee Busiah

**Affiliations:** ^1^ Faculty of Biology and Medicine, University of Lausanne, Lausanne, Switzerland; ^2^ Department of Pediatric, Neuchatel Regional Hospital, Neuchâtel, Switzerland; ^3^ Pediatric Endocrinology, Diabetology and Obesity Unit, Women-Mothers-Children Department, Lausanne University Hospital, Lausanne, Switzerland; ^4^ Pediatric Cardiology Unit, Women-Mothers-Children Department, Lausanne University Hospital, University of Lausanne, Lausanne, Switzerland; ^5^ Cardiology Unit, Heart-Vessels Department, Lausanne University Hospital, University of Lausanne, Lausanne, Switzerland; ^6^ Pediatric Ear, Nose and Throat Unit, Surgery Department, Lausanne University Hospital, University of Lausanne, Lausanne, Switzerland; ^7^ Clinical Chemistry Service, Laboratory Medicine and Pathology Department, Lausanne University Hospital, Lausanne, Switzerland; ^8^ Laboratory Medicine and Pathology Unit, Genetic Labs Department, Lausanne University Hospital, Lausanne, Switzerland; ^9^ Centre de Procréation Médicalement Assistée (CPMA), Lausanne, Switzerland; ^10^ Department of Pediatrics and Pediatric Endocrinology, Faculty of Medical Sciences, Medical University of Silesia, Katowice, Poland; ^11^ Endocrinology, Diabetology and Obesity Unit, Medicine Department, Lausanne University Hospital, Lausanne, Switzerland

**Keywords:** Turner syndrome, international guidelines, follow-up, transition, recommendations, care coordination, comorbidities

## Abstract

**Introduction:**

Turner syndrome association with multi-organ system comorbidities highlights the need for effective implementation of follow-up guidelines. We aimed to assess the adequacy of care with international guidelines published in 2007 and 2017 and to describe the phenotype of patients.

**Methods:**

In this multicenter retrospective descriptive cohort study, we collected growth and pubertal parameters, associated comorbidities, treatment, and karyotype in patients diagnosed at age <18 years between 1993 and 2022. We assessed age-appropriate recommendation follow-up (children, adolescents and adults) according to the 2007 guidelines if the last visit was before 2017 (18 recommendations) and the 2017 guidelines if the last visit was after 2017 (19 recommendations).

**Results:**

We included 68 patients followed at Lausanne University Hospital (n=64) and at Neuchatel Regional Hospital (RHNe) (n=4). 2.9% of patients underwent all recommended investigations.

Overall, 68.9 ± 22.5% and 78.5 ± 20.6% of the recommendations were followed, before and after 2017 respectively. High implementation rates were found for height, weight and BMI (100%), cardiac (80 to 100%) and renal (90 to 100%) imaging. Low implementation rates were found for Ear, Nose and Throat (ENT) (56.5%), skin (38.5%), dental (23.1%), ophthalmological (10%) and cholestasis (0 to 29%) assessments, depending on age and time of visit. In adults (n=33), the mean proportion of followed recommendations was lower before than after 2017: 63.5 ± 25.8% vs. 78.7 ± 23.4%, p=0.039.

**Conclusion:**

Growth parameters, cardiac and renal imaging are well followed. However, efforts should be made for dental, ENT, ophthalmological, skin and cholestasis assessments. Adequacy of follow-up improved with the quality of transition to adult care.

## 1 Introduction

Turner syndrome (TS), caused by the complete or partial absence of one of the two X chromosomes, is the most common sex chromosome disorder in females, affecting approximately 1 in 2,000 live-born females. Comorbidities of TS may involve the endocrine system (the main clinical features are short stature, hypogonadism due to ovarian dysgenesis, and thyroid disease), the cardiovascular system (congenital heart disease, aortopathy, vasculopathy, arterial hypertension) and neuropsychocognitive development (e.g. learning difficulties). Other possible manifestations include hearing loss, orthopedic disorders (hip dysplasia, scoliosis, osteoporosis), renal and urinary tract disorders, (metabolic syndrome (hypertension, insulin-resistance, diabetes, overweight) or autoimmune disorders (hypothyroidism, celiac disease). This explains the need for screening and lifelong multidisciplinary follow-up of these patients (1). As growth and pubertal disorders are the main complaints of patients with Turner syndrome, the pediatric endocrinologist is usually the first to start the workup. The transition of care to adult specialists will most often occur at the end of puberty.

International clinical practice guidelines for TS were first published in 2007 (2) and updated in 2017, with recommendations for care across the lifespan and covering all health issues and comorbidities (3). Based on these recommendations, the pediatric and adult endocrine units of the Lausanne University Hospital developed an in-house clinical care guideline in 2011. This document was upgraded in 2019 into a mobile, electronic health (m-health) tool called the TS health transition passport (**Supplementary Data Sheet 1**). The aim is to support patients’ understanding of their condition and improve effective transition to adult-oriented care.

The aims of our study were, first, to assess the adequacy of care according to published international recommendations in a tertiary center with pediatric and adult endocrine units (Lausanne University Hospital) and in a general hospital pediatric service (Neuchâtel Regional Hospital) and, second, to describe the clinical, biological and radiological phenotype of patients.

## 2 Methods

We conducted a retrospective study on patients affected with Turner syndrome.

### 2.1 Patients’ selection

We included patients with karyotype-confirmed TS, diagnosed before the age of 18 years and followed between 1993 and 2022 at the pediatric and adult endocrinology units of Lausanne University Hospital (CHUV) and the pediatric endocrinology unit of Neuchatel Regional Hospital (RHNe). No adult patients were followed at the RHNe. We excluded patients with less than 5% mosaic cells or with a written refusal.

### 2.2 Data collection

We collected clinical, radiological and biological data obtained during follow-up from medical records including karyotype, growth parameters, growth laboratory tests (IGF-1 and IGFBP-3 concentrations), growth treatment information and pubertal parameters.

Height was expressed as standard deviation (SD) using healthy female growth charts. Serum IGF-1 and IGFBP-3 were routinely assayed using IGF-1 and IGFBP-3 immunoassay kits: Nichols Institute Diagnostics from 1995 to 2005; Immulite by Siemens thereafter. Reference values were from Le Bouc Y for the Nichols Institute Diagnostics kit (4) and from Elminger et al. (5) for the Immulite kit.

We recorded comorbidities as follows: number of surgical treatments, presence of heart disease, hearing impairment, liver disease, dysthyroidism, renal disease, bone disease and celiac disease, transthoracic echocardiography (TTE) and cardiac magnetic resonance (CMR), abdominal-pelvic US, total body bone mineral density and laboratory tests such as creatinine, urea, anti-transglutaminase antibody, Thyroid Stimulating Hormone (TSH), free Thyroxine (free T4), anti-TPO antibody, glycated hemoglobin (HbA1C), Alanine Aminotransferase (ALAT), Aspartate Aminotransferase (ASAT), Gamma-Glutamyl Transpeptidase (γGT) and Alkaline phosphatase (ALP) concentrations.

### 2.3 Adequacy of follow-up

We defined the last visit as the last endocrinology consultation and if there was none, the last specialist consultation for patients who were no longer followed up in the pediatric unit. The last visit was the date for assessing adequacy of follow-up. We defined good adequacy of recommendation if ≥65% of patients followed it.

To assess the adequacy of follow-up, patients were divided into 2 groups: those whose last visit was before 2017 and those whose last visit was after 2017. Their follow-up was compared with the appropriate guidelines at the time, i.e. the 2007 guidelines and the 2017 guidelines, according to their age and pubertal status. The results were as follows: children, adolescents (girls with a Tanner stage S2 stage or higher -spontaneous or with estrogen therapy- and <18 years old), and adults.

### 2.4 Karyotype and cohort description

The diagnosis of TS was confirmed in all patients by karyotype using routine G-banding, including counting of at least 30 metaphases. We divided patients into 4 groups according to their karyotype: complete monosomy X (45,X); 45,X mosaicism (45,X/46,XX; 45,X/47,XXX; 45,X/46,XX/47,XXX; 45,X/46,XY); X structural rearrangement [45,X/46,X,del(Xp), 46,X,del(Xp); 45,X/46,X,del(Xq), 46,X,del(Xq); 45,X/46,X,i(Xq), 46,X,i(Xq); 45,X/46,X,r(X)], and Y structural rearrangement [45,X/46,X,idic (Y)]. We then described clinical and biochemical profiles according to karyotype.

### 2.5 Statistical analyses

Qualitative data were expressed as absolute number (percentage) and quantitative data as median (interquartile range - IQR) or as mean ± Standard Deviation (SD). We compared groups with Kruskall-Wallis, Chi^2^ or Fisher’s exact tests, using R statistical software. Values of *p* smaller than 0.05 were considered statistically significant.

This study was approved by the Local Ethics Committee (N°2021-00229).

## 3 Results

**Figure 1:**
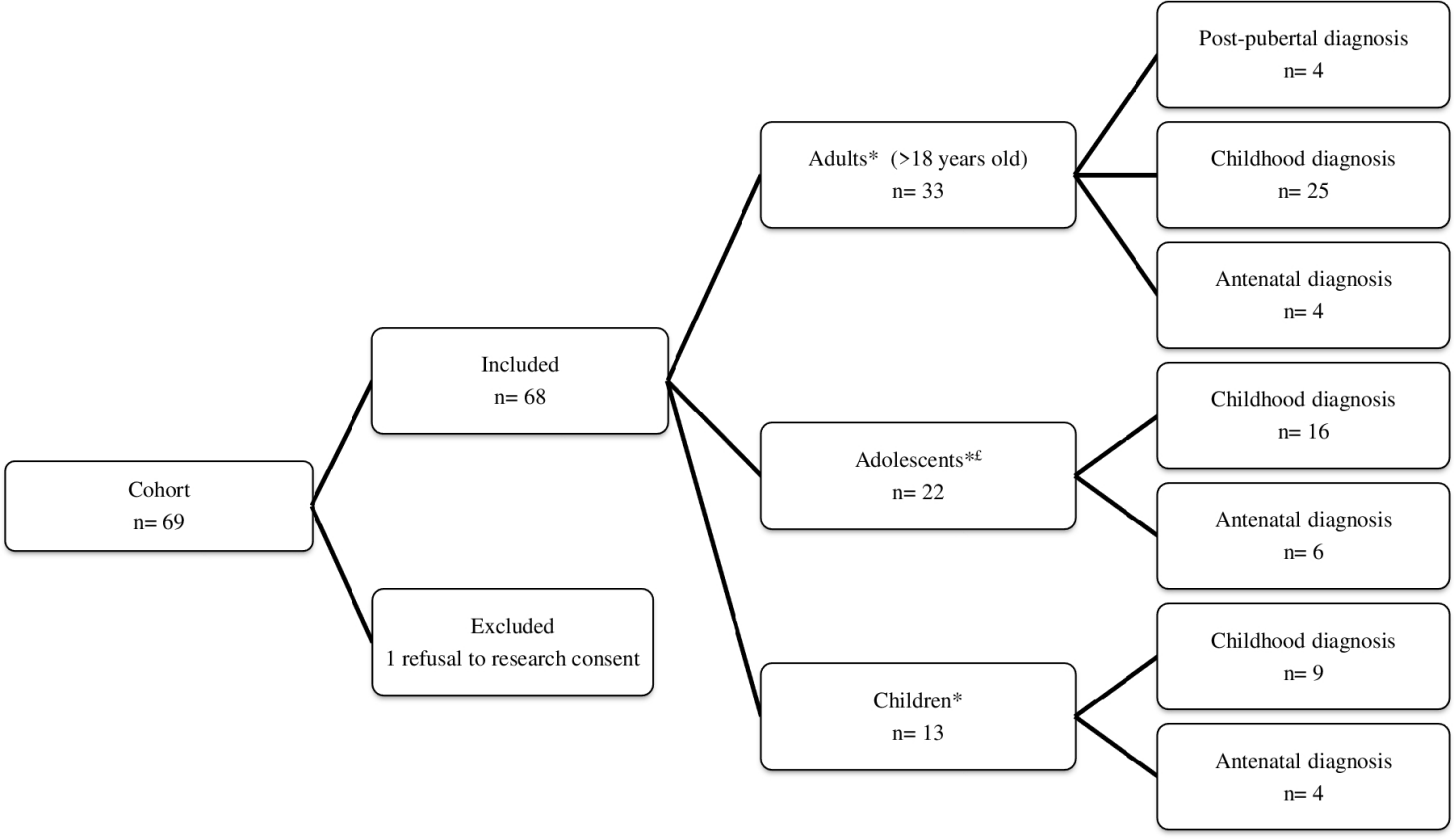
Patient flow chart. *Age at last visit: ^£^Girls with a Tanner S2 stage or above (spontaneously or with estrogen therapy) and aged below 18 years old.

We included 68 patients (**Figure 1**). Mean age at diagnosis was 6.3 ± 5.3 (range: 0 to 16.7) years. At last visit, there were 13 children, 22 adolescents and 33 adults. Mean age at last visit was 17.6 ± 7.5 (range: 1.2 to 40.5) years. We identified 17 patients (25%) who had their last visit before 2017 and 51 (75%) patients who had their last visit after 2017 (**Table 1**). All children had their last visit after 2017. In our clinic, we implemented 3 additional recommendations: IGFBP-3, thyroid US and bone age assessment, that are usually performed in children affected with growth retardation or dysthyroidism (**Supplementary Table 1**).

**Table 1:** Adequacy of recommendations of care according to the 2007 and the 2017 international guidelines.

Items	2007 recommendations^1^				2017 recommendations^2^				
	Monitoring	Adolescents	Adults	P-value ^3^	Monitoring	Children	Adolescents	Adults	P-value ^3^
Number of patients total n=68 (100%)		7	10	NA		13	15	23	NA
Clinic (%)									
Height/Weight/BMI	Not mentioned	NA	NA	NA	Annually	100	100	100	1
Fertility counseling	At least once	100	90	1	At least once	38.5	93	91	<0.001
ENT and audiology	Each 1-5 Y	57	80	0.6	Each 3 Y (children and adolescents), each 5 Y (adults)	77	80	56.5	0.2
Ophthalmology	Between 0-4 Y (If age > 1Y)	0	10	1	At least once	61.5	67	78	0.5
Skin examination	Not mentioned	NA	NA	NA	Annually	38.5	67	70	0.2
Dental specialist	At least once	43	30	0.6	At least once	23	53	48	0.2
Biology (%)									
Fasting glucose	Annually (> 10Y)	100	90	1	Annually (> 10Y)	100^4^	87	91	1
HbA1c	Annually (> 10Y)	100	40	**0.035**	Annually (> 10Y)	100^4^	87	61	0.2
IGF-1 (on GH treatment)	Not mentioned	NA	NA	NA	Annually (< 18 Y)	70^5^	64^5^	NA	1
TSH and Free T4	Annually (> 4 Y)	86	70	0.6	Annually (no starting age)	69	100	83	0.066
Anti-TPO antibody	Not mentioned	NA	NA	NA	If dysthyroidy	No dysthyroidy	100 ^7^	100 ^7^	1
Celiac screen	Each 2-5 Y (> 4Y)	86	60	0.4	Each 2 Y (from 2 to 18 Y)	69	93	NA^6^	0.2
Total cholesterol, HDL, LDL, TG	Annually (> 10 Y)	86	70	0.6	Annually (> 18 Y if cardiovascular risk factors^8^)	NA	NA	87	NA
Creatinine/Urea	Each 1-2 Y	29	40	1	Not mentioned	NA	NA	NA	NA
ASAT/ALAT	Annually (> 10Y)	43	60	1	Annually (> 10Y)	100^4^	80	83	1
γGT/ALP	Annually (> 10Y)	29	20	0.6	Annually (> 10Y)	0^4^	27	52	0.2
Imaging (%)									
Renal US	At least once	100	90	1	At least once	100	93	96	1
ECG	At least once	86	70	0.6	At least once	92	100	91	0.6
TTE or CMR	Each 5-10 Y^9^	100	80	0.5	Each 5 Y	100	100	87	0.2
Bone mineral density	No specific time interval (>18 Y)	NA	30	NA	Each 5 Y (> 18 Y)	NA	NA	78	NA

ALAT, Alanine Aminotransferase; ALP, Alkaline phosphatase; ASAT, Aspartate Aminotransferase; BMI, Body Mass Index; CMR, Cardiac magnetic resonance; ECG, Electrocardiogram; ENT, Ear, Nose and Throat; γGT, Gamma-Glutamyl Transpeptidase; HBA1C, Glycated hemoglobin; IGF-1, Insulin-like Growth Factor 1; NA, Non-applicable; T4, Thyroxin; TG, Triglyceride; TSH, Thyroid Stimulating Hormone; TTE, Transthoracic echocardiography; US, Ultrasound; Y, years.

All children had their last follow up visit after 2017.

^1^From Bondy et al. (2007); ^2^From Gravholt et al. (2017);^3^The p-values were calculated across groups: “Children”, “Adolescents” and “Adults” with the Fisher’s Exact Test; ^4^1 patient > 10 Y; ^5^10 children and 11 adolescents with GH treatment, ^6^To do when suggestive symptoms ^7^2 adolescents and 10 adults with dysthyroidsm, ^8^Considered to be present in all adults in our cohort, ^9^if normal anatomy, otherwise according to the opinion of the cardiologist.

### 3.1 Follow-up adequacy

Overall, 2.9% (n=2/68, one child and one adult last seen after 2017) of the patients underwent all recommended investigations. The overall mean proportion of recommendations followed was 76.1 ± 21.4%: 68.9 ± 22.5% of the 2007 recommendations for patients last seen before 2017 and 78.5 ± 20.6% of the 2017 recommendations for patients last seen after 2017 (**Table 1** and **Supplementary Table 1**).

#### 3.1.1 Adequacy of follow-up according to recommendations

The recommendations with the highest implementation rate were height, weight and BMI (100%), and cardiac (range: 80 to 100%) and renal (range: 90 to 100%) imaging. The recommendations with the lowest implementation rate were bone mineral density (in adults last seen before 2017: 30%), skin examination (in children: 38.5%), ENT (in adolescents last seen before 2017: 57% and in adults last seen after 2017: 56.5%), ophthalmological (in adolescents and adults last seen before 2017: respectively: 0% and 10%, and children: 61.5%) and dental consultations for the whole cohort (**Table 1**). Liver function biomarkers were often not assayed, especially ASAT and ALAT for adolescents last seen before 2017 (43%), and γGT and ALP for the whole cohort (**Table 1**).

We found a difference for HbA1C between adolescents and adults last seen before 2017 (100% vs. 40% p=0.035) and for fertility counseling among all patients last seen after 2017 (38.5% of children, 93.3% of adolescents and 91.3% of adults, p<0.001).

#### 3.1.2 Adequacy of follow-up according to age

In children (n=13), the overall mean proportion of recommendations followed was 75.5 ± 19.1% (**Table 1**). We found good adequacy (i.e., ≥65% of recommendations followed) for 11/16 (69%) of the recommendations in children. In adolescents (n=22), we found no difference between overall followed recommendations for patients last seen before 2017 compared to patients last seen after 2017 (76.6 ± 15.1% vs. 80.9 ± 18.3%, p=0.306). We found good adequacy for 9/15 (60%) of the recommendations for adolescents last seen before 2017 and 14/17 (82%) of the recommendations for adolescents last seen after 2017. In contrast, in adults (n=33), the mean proportion of overall followed recommendations was lower before than after 2017: 63.5 ± 25.8% vs. 78.7 ± 23.4%, respectively, p=0.039. We found good adequacy for 8/16 (50%) of the recommendations for adults last seen before 2017 and for 13/17 (76%) of the recommendations for adults last seen after 2017.

All children and adolescents had cardiac imaging, whereas 20% and 13% of adults last seen before and after 2017 respectively did not have any cardiac imaging; the difference was not significant.

### 3.2 Growth, puberty and comorbidities

We then compared patients’ clinical, biological and radiological findings according to karyotypes, that were as follows: monosomy 45,X (n=24, 35.3%); 45,X mosaicism (n=18, 26.5%); X chromosome structural rearrangement (n=24, 35.3%); and Y chromosome structural rearrangement (n=2, 2.9%).

Height and height velocity at diagnosis were significantly different across the groups (p=0.031 and p=0.019, respectively) (**Table 2**). Nevertheless, final height was similar across the groups (p=0.106).

**Table 2:** Anthropometric characteristics according to karyotype.

	Total	Complete Monosomy X	45,X Mosaicism	X Structural Rearrangement	Y Structural Rearrangement	p-value*
Turner syndrome *n* (%)	68 (100%)	24 (35.3%)	18 (26.5%)	24 (35.3%)	2 (2.9%)	NA
At diagnosis						
Age at diagnosis, years	5.3 (0.8 to 9.9)	4.9 (0.0 to 10.5)	2.15 (0.0 to 6.1)	8.1 (4.5 to 11.2)	9.0 (6.8 to 11.3)	0.015
Height at diagnosis, SD	-2.2 (-2.5 to -1.6)	-2.3 (-2.4 to -1.6)	-1.8 (-2.1 to -0.6)	-2.4 (-3.0 to -1.9)	-2.5	0.031
Height velocity at diagnosis, SD	-1.1 (-2.2 to 1.3)	-2.4 (-2.7 to -2.1)	1.9 (0.3 to 2.0)	-0.3 (-1.0 to 2.0)	Not available	0.019
At last visit						
Age at last visit, years	17.9 (13.6 to 21.7)	19.4 (16.5 to 25.9)	17.6 (12.7 to 21.8)	16.2 (12.8 to 19.9)	12.1 (10.7 to 13.6)	0.084
Height at last visit, SD	-1.6 (-2.3 to -1.1)	-1.8 (-2.2 to 1.4)	-0.8 (-1.6 to -0.2)	-2.2 (-2.7 to -1.3)	-0.7 (-1.1 to -0.2)	0.002
Final height, SD	-1.7 (-2.4 to -1.1)	-1.8 (-2.2 to -1.3)	-1.4 (-1.9 to -0.2)	-2.3 (-2.8 to -1.3)	Not available	0.106

Quantitative data are expressed as median and interquartile range (IQR).

SD, Standard Deviation.

*The p-values were calculated across 3 groups: “Complete Monosomy X”, “45,X Mosaicism” and “X Structural Rearrangement” with the Kruskal-Wallis test.

Among the 86.8% of patients who had GH treatment, height and height velocity at the start of GH were significantly different across the groups (p=0.017 and 0.006 respectively) (**Table 3**). One year after the start of GH treatment, height velocity was no longer different across the groups (p=0.971) while the height remained significantly different (p=0.021). Bone age delay of more than 1 year was found in 23/46 (50%) of patients at the start of GH treatment and in 12/46 (26.1%) of patients at the end of GH. In these 12 patients, GH therapy was discontinued despite the bone delay, because of patients’ willingness.

**Table 3:** Main clinical and laboratory characteristics on growth hormone treatment according to karyotype.

	Total	Complete Monosomy X	45,X Mosaicism	X Structural Rearrangement	Y Structural Rearrangement	p-value*
Growth hormone treatment n (%)	59 (86.8%)	23 (95.8%)	12 (66.7%)	22 (91.7%)	2 (100%)	0.094
At start of GH						
Age, years	7.4 (5.0 to 10.6)	6.5 (4.5 to 10.5)	7.0 (5.4 to 9.8)	8.9 (6.0 to 11.2)	9.07 (6.9 to 11.3)	0.554
Starting dose of GH, µg/kg/day	34.0 (22.9 to 41.4)	35.71 (22.9 to 41.4)	38.6 (24.3 to 41.4)	25.7 (22.9 to 37.1)	31.4 (30.0 to 31.4)	0.584
Height, SD	-2.3 (-2.6 to -1.6)	-2.4 (-2.5 to -2.0)	-1.5 (-2.1 to -1.3)	-2.4 (-3.0 to -2.1)	-1.6 (-2.1 to -1.2)	0.017
Height velocity, SD	-1.4 (-1.9 to 0.0)	-1.8 (-2.8 to -1.6)	0.0 (-0.2 to 1.9)	-1.4 (-2.1 to -1.1)	Not available	0.006
IGF-1, SD	-0.8 (-1.4 to 0.3)	-1.2 (-2.2 to -0.3)	-0.5 (-1.2 to 0.3)	-0.8 (-1.3 to 0.1)	-0.6	0.393
IGFBP-3, SD	0.5 (-0.0 to 1.3)	0.3 (-0.2 to 0.8)	1.1 (0.2 to 1.8)	0.5 (0.2 to 0.8)	-1.8	0.382
One year after start of GH						
Height, SD	-1.9 (-2.2 to -1.1)	-1.8 (-2.3 to -1.3)	-1.0 (-1.8 to -0.7)	-2.1 (-2.5 to -1.8)	-1.4 (-1.7 to -1.0)	0.021
Height velocity, SD	1.4 (0.6 to 2.5)	1.5 (0.3 to 3.8)	1.7 (1.0 to 2.1)	1.4 (0.7 to 2.3)	1.1 (1.1 to 1.1)	0.971
At maximum dose of GH						
Maximum dose of GH, µg/kg/day	45.7 (42.9 to 50.0)	47.1 (47.1 to 58.6)	45.7 (42.9 to 50.0)	42.9 (42.9 to 48.6)	31.4	0.060
Age, years	11.6 (8.9 to 13.4)	12.0 (8.6 to 14.0)	11.5 (9.7 to 12.6)	10.8 (8.9 to 13.3)	15.0	0.895
IGF-1, SD	0.9 (0.1 to 1.8)	0.3 (-0.5 to 1.1)	0.7 (0.3 to 1.2)	1.4 (0.9 to 2.2)	0.6	0.018
IGFBP-3, SD	0.9 (0.2 to 1.3)	0.9 (0.1 to 1.3)	1.2 (0.7 to 1.4)	0.8 (0.4 to 1.2)	0.2	0.695
At the end of GH						
Age, years	15.6 (14.9 to 17.0)	16.3 (15.5 to 17.4)	14.9 (14.7 to 15.2)	16.6 (14.8 to 17.2)	Non applicable	0.011
Dose of GH, µg/kg/day	42.9 (41.4 to 45.7)	42.9 (41.4 to 45.7)	41.4 (40.0 to 45.7)	41.4 (38.6 to 45.7)	Non applicable	0.458
GH duration, years	8.0 (4.6 to 10.1)	8.9 (6.0 to 11.2)	8.0 (5.2 to 9.8)	7.2 (2.6 to 8.1)	Non applicable	0.192

Quantitative data are expressed as median and interquartile range (IQR).

GH, Growth Hormone; IGF-1, Insulin-like Growth Factor One; IGFBP-3, Insulin-like Growth Factor Binding Protein-3; SD, Standard Deviation.

*The p-values were calculated across 3 groups: “Complete Monosomy X”, “45,X Mosaicism” and “X Structural Rearrangement” with the Kruskal-Wallis test.

Puberty was spontaneous in 3 (13.6%) 45,X patients (**Table 4**). Puberty was induced in 28 (50.9%) patients, mainly in the 45,X group (p<0.001), and after 12 years of age for 20/28 (71.4%) patients. 40 patients received estrogen therapy, 29 by oral administration route and 11 by transdermal administration route.

**Table 4:** Clinical and biological puberty characteristics according to karyotype.

	Total	Complete Monosomy X	45,X Mosaicism	X Structural Rearrangement	Y Structural Rearrangement	p-value*
Spontaneous puberty onset n (%)	27 (49.1%)	3 (13.6%)	12 (85.7%)	12 (66.7%)	0 (0%)	<0.001
Induced puberty onset n (%)	28 (50.9%)	19 (86.4%)	2 (14.3%)	6 (33.3%)	1 (100%)	
Age at the onset of puberty, years	12.0 (11.1 to 12.7)	12.3 (11.6 to 13.5)	10.3 (10.2 to 11.6)	12.1 (11.8 to 13.0)	13.9	0.001
E2 therapy n (%)	40 (72.7%)	22 (100%)	3 (21.4%)	14 (77.8%)	1 (100%)	<0.001
Age at the start of E2, years	12.8 (12.1 to 15.0)	12.5 (11.9 to 14.6)	11.8 (11.7 to 14.2)	13.8 (12.8 to 15.4)	13.9	0.104
Starting dose of E2,μg/day	3 (2 to 20)	3 (2 to 10)	4 (3 to 502)	6 (2 to 813)	5	0.260
Progesterone therapy n (%)	32 (58.2%)	19 (86.4%)	2 (14.3%)	11 (61.1%)	0 (0%)	<0.001
Age at the start of Progesterone, years	16.5 (14.5 to 17.4)	15.8 (14.5 to 17.4)	15.4 (14.9 to 16.0)	17.1 (15.8 to 17.4)	Non applicable	0.524
Starting dose of Progesterone, mg/day	10	10	10	10	Non applicable	0.575
FSH at start of E2 or at start of puberty, U/l	27.4 (4.0 to 87.5)	81.9 (31.8 to 121.6)	3.9 (1.9 to 8.9)	47.0 (7.5 to 82.7)	98.6	0.001
LH at start of E2 or at start of puberty, U/l	13.1 (1.3 to 24.2)	23.7 (15.3 to 28.5)	2.0 (0.5 to 6.5)	12.7 (1.0 to 21.7)	12.6	0.010
AMH, pmol/l	13.1 (9.0 to 22.4)	8.8	16.9 (9.7 to 23.1)	12.0 (8.0 to 28.5)	Non applicable	0.777**
Spontaneous menstruation n (%)	24 (46.2%)	2 (9.5%)	12 (85.7%)	10 (62.5%)	0 (0%)	<0.001
Induced menstruation n (%)	24 (46.2%)	16 (76.2%)	2 (14.3%)	5 (31.3%)	1 (100%)	
Age at first menstrual period, years	14.4 (13.2 to 15.5)	15.7 (14.4 to 16.9)	12.7 (12.1 to 13.6)	14.4 (13.3 to 15.3)	Non applicable	<0.001
Total body bone densitometry, Z-score	-0.1 (-1.3 to 0.6)	-0.3 (-1.2 to 0.6)	1.3 (-0.1 to 1.8)	-0.3 (-1.4 to 0.2)	Non applicable	0.221
Age at bone densitometry, years	18.9 (14.9 to 23.3)	23.3 (18.9 to 27.2)	18.6 (14.7 to 22.1)	16.1 (12.9 to 21.4)	Non applicable	0.055

Quantitative data are expressed as median and interquartile range (IQR).

AMH, Anti-Müllerian Hormone; E2, Oestrogen; FSH, Follicle Stimulating Hormone; LH, Luteinizing hormone.

*These p-values were calculated across 3 groups “Complete Monosomy X”, “45,X Mosaicism” and “X Structural Rearrangement” with the Kruskal-Wallis test.

**These p-values were calculated across 2 groups “45,X Mosaicism” and “X Structural Rearrangement”. AMH was detectable in one patient in the “Complete Monosomy X” group.

Heart disease, kidney disease, celiac disease and surgery were significantly different according to their karyotype (**Table 5**). Osteopenia, defined as a BMD Z-score <-1, was present in 7/24 (29%) patients.

**Table 5:** Frequency of co-morbidities and associated features according to karyotype.

	Total	Complete Monosomy X	45,X Mosaicism	X Structural Rearrangement	Y Structural Rearrangement	p-value***
Heart disease n (%)	31 (45.6%)	16 (66.7%)	8 (44.4%)	7 (29.2%)	0 (0%)	0.011
Hearing impairment n (%)	25 (36.8%)	11 (45.8%)	5 (27.8%)	8 (33.3%)	1 (50%)	0.377
Hypothyroidism n (%)	15 (22.1%)	7 (29.2%)	4 (22.2%)	4 (16.7%)	0 (0%)	0.576
Renal disease n (%)	13 (19.1%)	8 (33.3%)	2 (11.1%)	2 (8.3%)	1 (0%)	0.049
Bone disease* n (%)	6 (8.8%)	2 (8.3%)	1 (5.6%)	3 (12.5%)	0 (0%)	0.867
Celiac Disease n (%)	4 (5.9%)	4 (16.7%)	0 (0%)	0 (0%)	0 (0%)	0.016
Liver disease** n (%)	3 (4.4%)	1 (4.2%)	0 (0%)	2 (8.3%)	0 (0%)	0.771

*Bone disease referred to osteopenia, Léri-Weill dyschondrosteosis and cartilaginous protrusion of the ribs.

**Liver diseases were steatohepatitis with hepatomegaly for a 16 years and a 20 years old patient and inflammatory liver disease for a 30 years old patient.

***The p-values were calculated across 3 groups “Complete Monosomy X”, “45,X Mosaicism” and “X Structural Rearrangement” with the Chi^2^ test or with Fisher’s exact test when Chi^2^ test was not applicable.

## 4 Discussion

In this cohort of children, adolescents and adults patients with TS, a small minority of patients had a complete follow-up according to the international guidelines. However, the most important and potentially serious comorbidities were well followed-up, especially growth parameters, cardiac assessment and renal ultrasound. Adequacy of follow-up improved with quality of transition to adult care.

Four studies evaluated the adequacy of care according to international guidelines: three compared the adequacy of follow-up with the 2007 guidelines, and one with the 2017 guidelines (6–9). Two studies from France and Poland found that less than 5% of adult patients received all the medical investigations recommended in the 2007 guidelines. They showed that liver enzymes were often not assayed (6, 7). The prevalence of liver disease is higher in adults with TS, especially with elevated γGT rather than transaminases (10). Reported complications include non-alcoholic steatohepatitis, hepatic architectural changes such as cirrhosis, and biliary lesions such as sclerosing cholangitis (1, 11). An American study evaluated the medical care of girls with TS compared to the 2007 guidelines. In our study, adherence to recommendations was higher for follow-up of lipid levels, liver enzymes, blood glucose, thyroid function, ENT assessments, fertility counselling, celiac screening and bone mineral density and cardiac imaging, depending on the study (6–8). However, celiac screening, ENT and ophthalmological assessments were lower than in the study published by Hoag and colleagues (9). This suggests an improvement in the management of patients with TS. There is still room for improvement in care coordination. The implementation of new models of care coordination could help (12).

We focused on more recommendations from the 2007 and 2017 guidelines than these studies, especially dental, eye and skin examinations. Lack of compliance with these follow-up visits can lead to reduced quality of life. This highlights the importance of awareness among clinicians. Dermatological screening aims to detect lymphedema, dermatitis, eczema, psoriasis and multiple pigmented nevi (1, 13). Eye disorders include high rates of strabismus, visual impairment such as myopia, or sight-threatening abnormalities such as papilledema (14). The time interval recommended by the 2007 guidelines for seeing an ophthalmologist was very restrictive. This might explain the poor follow-up adequacy of ophthalmic consultations for patients with last visit before 2017. Dental disorders include a wide range of manifestations from micrognathia to abnormal dental development (1). The fact that dental consultations are not covered by the Swiss National Health Insurance may explain the low number of dental consultations in our cohort. The distribution of karyotypes in our cohort showed slightly higher proportion of X structural rearrangement than previously published (15). The comorbidities and their distribution between the different karyotypes in our cohort were globally consistent with the literature (1, 16–18). In our study, we found lower proportions of heart and liver diseases but similar proportions of thyroid and celiac diseases (15). Nevertheless, the number of comorbidities should be correlated with the adherence to recommended follow-up.

All recommendations, except HbA1c for patients last seen before 2017 and fertility counselling for patients last seen after 2017, were monitored equally between children, adolescents and adults. This may reflect the structured transition clinic between pediatric and adult endocrinological care that we have developed at the CHUV. This transition endocrine clinic has improved, as suggested by a better follow-up of adults last seen before 2017 compared to those last seen after 2017. As all RHNe patients are children or adolescents, they did not have transition. Our group has shown the usefulness of integrating transition passports as a usable, understandable health tool for patients and physicians, to reduce gaps in transition from pediatric to adult-oriented care (19). Our study suggests that the CHUV pediatric and adult TS transition passport could provide patients with a better understanding of their follow-up, of treatment, fertility care and comorbidities.

GH treatment followed the 2017 guidelines in terms of starting age, doses and IGF-1 monitoring. Estrogen treatment was started at low doses and increased over 2 to 3 years as recommended. However, the majority of patients started later than recommended. This discrepancy may be explained by the fact that only five patients started treatment after the publication of the 2017 guidelines (3). Late initiation of estrogen therapy can be detrimental to bone and uterine health (20). Data are consistent with no change in adult height when low-doses estrogen is started before the age of 12, as recommended.

The strengths of our study include the comparison of long-term medical follow-up of girls and women with TS between the two published international guidelines. Studies on the adequacy of medical follow-up in girls and women with TS are limited (6–9), and most have compared the adequacy of follow-up with the 2007 guideline. We divided our cohort into 3 different age groups, as in the study by Hoag and colleagues (9). We also included additional important recommendations such as dental and ophthalmological consultations and skin examinations, which are rarely investigated, and we could perform a karyotype in all patients. This study looked at patients with TS followed at a large university center (CHUV) with many experienced specialists, including cardiologists, radiologists, ear, nose and throat specialists, adult endocrinologists, and others. As a result, follow-up of these patients may not be the same throughout Switzerland. Moreover, in other studies, most adult patients with TS were followed up by general practitioners, who were sometimes unaware of the TS diagnosis. For this reason, we developed a patient oriented electronic health (m-health) TS health transition passport, to avoid loss of medical information (**Supplementary Data Sheet 1**).

A limitation of our study is its retrospective design, which is justified by the rarity of the disease. However, we minimized missing data through in-depth analysis of medical records.

Our study highlights the importance of improving awareness among patients themselves and primary care physicians of the broad spectrum and variability of TS presentation at different ages. We should aim to reduce health inequalities by making multidisciplinary clinics and comprehensive care available and accessible. It is also important to ensure adequate medical and social support for transition of young adults and care of adults with TS. We also should involve the patient, who gains autonomy and responsibility for her health care during adolescence and young adulthood. Our TS education program, launched in 2011 and improved in 2019, aims to address these challenges. We aim to serve as a regional resource for the community and for physicians in our community.

In conclusion, complete guideline adherence in TS patient care and follow-up should be improved, especially in bone mineral density, liver, ophthalmic, ENT, dermatological and dental assessment. Our results open a field for possible future research on patient education and healthcare organizations: how to understand the lack of awareness in TS, how to improve structural problems, how to implement a complete work-up or how to spread these guidelines among non-endocrinologists, especially pediatricians and general practitioners. A better follow-up has already been observed compared to the studies before 2017, which makes us optimistic for the future.

## Data availability statement

The original contributions presented in the study are included in the article/**Supplementary Material**, further inquiries can be directed to the corresponding author/s.

## Ethics statement

The studies involving human participants were reviewed and approved by Commission cantonale (VD) d’éthique de la recherche sur l’être humain (CER-VD). Written informed consent from the participants’ legal guardian/next of kin was not required to participate in this study in accordance with the national legislation and the institutional requirements.

## Author contributions

JL collected the data, performed the data analysis and interpretation, performed the statistical analysis, wrote, and critically revised the manuscript. KB designed the study, was responsible for the data analysis and interpretation, and for the statistical analysis, wrote, and critically revised the manuscript. NV and MH interpreted the data and critically revised the manuscript. SS-V, MA, TB, IR, NS, TR, SF, NP, MH, and KB followed the patients, PB was responsible for hormonal assay, FB was responsible for genetic investigation. AG provided feedback on the study design. All authors contributed to the article and approved the submitted version.
