# Supplementary material for: Turner syndrome: skin, liver, eyes, dental and ENT evaluation should be improved
Source: Front Endocrinol (Lausanne). 2023 Jul 25;14:1190670. doi: 10.3389/fendo.2023.1190670 (PMC10408677; doi:10.3389/fendo.2023.1190670)
Supplement: Supplementary file 1 [file DataSheet_1.pdf]

Turner Syndrome

A guide for patients with Turner syndrome

canton de vaud
LIBERTÉ ET PATRIE

CHUV

The syndrome in brief
Birth and Infancy
Childhood
Puberty
Young adult
Adult
Tests
Child - Traitement
Adult - Traitement
Fertility
Results
My follow up
My treatment
My contacts

The syndrome in brief

What is Turner syndrome ?

Turner syndrome is a rare genetic condition (1 in 2,500 female newborns) that often results in short stature and ovarian failure. Other manifestations may involve abnormalities of the heart and kidneys and particularities of the face and limbs. There is also an increased risk of subsequent acquired diseases. This syndrome is caused by the complete or partial absence of one of the two X chromosomes in women.

diagnosis: karyotype

Usually, women have two X chromosomes; their chromosomal count is 46, XX. Men have one X and one Y chromosome; their chromosomal count is 46, XY.

Turner syndrome is due to the total or partial loss of one X chromosome in the female fetus. Karyotyping is a test that involves analyzing chromosomes. The test can either be carried out while the baby is inside the womb (by taking a sample of amniotic fluid), or after birth by taking a sample of the baby's blood or tissue.

In about 50% of cases, karyotyping will reveal the total loss of one X chromosome, called 45, X monosomy.

In 20% of cases, the X chromosome is lost in only some of the body's cells. There are, therefore, both abnormal and normal cells: this is called mosaicism. Other forms involve structural defects of the X chromosome.

Characteristics of Turner syndrome

Females with Turner syndrome often have a wide range of characteristics and associated conditions.

These characteristics and conditions can be :

- Stunted growth and short stature
- Delayed or absence of puberty
- Heart abnormalities (like aortic coarctation or abnormalities of the valves such as the bicuspid aortic valve)
- High blood pressure (hypertension)
- Kidney and urinary tract problems (horseshoe-shaped kidney, reflux)
- Disorders of ears (frequent ear infections, possible sensorineural hearing loss)
- Orthodontic disorders (ogival palate, small mandible)
- Underactive or hyperactive thyroid gland (hypothyroidism or hyperthyroidism) and metabolic disorders such as diabetes
- Lymphoedemas

Most girls with Turner syndrome have good language and reading skills. However, some have behavioral, social, and specific learning difficulties, such as arithmetic. They may also experience emotional immaturity or a lack of self-confidence.

diagnosis/follow-up

Diagnosis is often made at birth, during childhood or adolescence, or rarely in adulthood. Diagnosis is confirmed by chromosome analysis (karyotype). Lifelong multidisciplinary follow-up is required.

Insurance

Specific insurance issues need to be discussed with the caring team.

At birth

At birth

At birth, the newborn with Turner syndrome may be completely normal or present one or more clinical signs, such as being small for gestational age, lymphedema on the backs of the hands and feet, or cardiac or renal malformations. Typical clinical signs may also be present, such as characteristic eyes (epicanthus, drooping eyelids called ptosis), a short, wide neck (webbed neck, called pterygium colli), a broad chest, and widely spaced nipples, a low hairline, upturned or spoon-shaped nails.

Diagnosis

An abnormality of the prenatal karyotype may be known if an amniocentesis is performed during pregnancy or if a cardiac malformation is detected during the pregnancy ultrasound.

Chromosome analysis (karyotype) after birth should confirm the diagnosis.

As soon as the diagnosis is made, malformations or associated abnormalities (heart, kidneys, audiogram) are investigated (see initial workup).

Management at birth depends on associated conditions (see initial assessment).

Childhood

Childhood

Most children with Turner syndrome develop normally. Short stature is present in 98% of cases, leading to consultation. Adult height is around 145 cm without growth hormone treatment.

Some children may also have :

- Recurring middle ear infections (otitis media) and glue ear.
- Dental problems (small chin called micrognathia, high arched palate)
- Bone disorders (abnormal curvature of the spine called scoliosis, congenital hip dislocation)
- Eye disorders (a squint called strabismus)

Other organs may also be affected: :

- The cardiovascular system: In around 30% of cases, there is a heart defect, particularly a narrowing of the main blood vessel in the heart, the aorta (coarctation of the aorta) or the aortic valve (bicuspid aortic valve). Early-onset hypertension is also common.

- Kidneys: :There may be a single kidney, a horseshoe kidney (the two kidneys are joined in a U-shape), or malformations of the urinary tract in around 20-30% of cases. These anomalies can lead to urinary tract infections and high blood pressure.

Learning difficulties, shyness, or suffering due to short stature may sometimes require educational or psychological support

If the diagnosis is made in childhood, the search for associated conditions (heart, kidney, audiogram) is recommended as soon as possible (see initial assessment).

Puberty

Puberty

Puberty occurs when the ovaries are stimulated by the pituitary hormones LH and FSH. Ovaries are the pair of female reproductive organs that produce eggs and sex hormones. During puberty, a girl's ovaries usually begin to produce the sex hormone estrogen, which leads to breast development, peak growth, bone mineralization, and maturation of the uterus. Once fully mature, ovaries will produce progesterone. This hormone triggers the start of periods.

In girls with Turner syndrome, the ovaries do not develop properly and may appear as fibrous bands.

The consequences of insufficient ovarian function can be :

- Absent sexual development or fully developed female breasts
- Absence of menstrual periods (amenorrhea)
- Reduced bone mass, leading to brittle bones (osteoporosis)
- Problems with body image or self-esteem

The diagnosis may be suspected in adolescence in the presence of the above signs, and confirmed by a karyotype.

A search for malformations and associated conditions (heart, kidney, audiogram, autoimmune disease, metabolic disorders) and a multidisciplinary follow-up will be initiated (see initial assessment).

Young adult

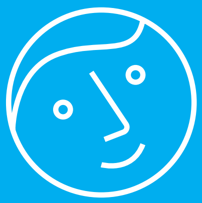

YOUNG ADULT

The period between the ages of 16 and 25 years is a complex phase of development, during which young people gradually acquire independence, social skills, and self-esteem. They discover their sexuality and search for their own identity.

The young women might consult for amenorrhea (absence of menstruation) and possibly infertility, which will lead to the diagnosis.

For girls affected with Turner Syndrome already followed up, the pediatric multidisciplinary team hands over to an adult multidisciplinary team during this period (transition period). Psychological support may be indicated.

In addition to the support provided by the care team, patients may be able to meet with other support groups or patients.

Adult

Adult

Adult women affected with Turner syndrome can live normally with appropriate treatment.

As ovarian function might be absent, hormone replacement therapy (estrogen and progesterone) is indicated to achieve proper estrogen impregnation of the genital tract. Estrogens are also beneficial for bone health and general well-being. Infertility is associated with ovarian insufficiency.

Multidisciplinary follow-up is recommended to monitor hormone treatment and detect any associated conditions:

- Cardiovascular defects
- Decreased bone mineralization with brittle bones (osteoporosis)
- Metabolic disorders such as diabetes or obesity
- Autoimmune diseases affecting the thyroid gland or celiac disease
- Hearing loss

A healthy lifestyle with a balanced diet and regular physical activity is recommended.

If ovarian function is preserved, follow-up by a fertility specialist is recommended. For other patients, appropriate management should be proposed (see infertility treatment).

Treatment at birth, for children, and teenagers

Treatment at birth

- Treatment at birth depends on the associated malformations (see initial assessment). Surgical interventions may be necessary for some heart or kidney defects.
- Most newborns do not require treatment.
- Growth monitoring is started

TREATMENT IN CHILDREN AND ADOLESCENTS

- Spontaneous adult height varies between 142 and 147 cm without treatment. For this reason, treatment of growth retardation with growth hormone (hGH) is often indicated. In over 50% of cases, treatment with growth hormone results in an adult height of over 150 cm. Growth hormone is a substance produced naturally by a gland at the base of the brain (the pituitary gland).
- Growth hormone therapy consists of daily subcutaneous injections. It can be administered at home by the child and her parents. Treatment is continued until the girl's skeleton reaches a stage similar to that of a 14-year-old child. This is known as the 14 years old bone age, which is determined by X-rays.
- Specific treatment depends on associated malformations (see initial assessment).
- In some cases, school support may be helpful.
- Estrogen and progesterone, normally produced by ovaries, are essential. Estrogen replacement therapy is usually started around the time of normal puberty. This treatment ensures good pubertal growth, good development of pubertal signs, and appropriate bone mineralization (osteoporosis prevention). Estrogens are given in progressive doses. In most cases, estrogen is administered as a transdermal patch. Tablets are also available. Treatments are tailored to the needs of the adolescent.
- In rare cases, fertility preservation treatment may be proposed (see fertility).
- Treatment of recurrent ear infections and screening for hearing loss is important. Hearing aids may be needed because hearing loss can interfere with schooling.
- Any problems with body image or self-esteem can be discussed with caregivers or a psychologist.
- Any associated health disorders, such as autoimmune or metabolic disorders, should be monitored:
  - Endocrine disorders (diabetes, hypothyroidism) may need hormonal treatment
  - Celiac disease is treated by following a specific gluten-free diet
  - Hypertension must be managed with an appropriate diet and medication
  - Orthopedic treatments (surgical and non-surgical) may also be required
- Regular physical activities and a healthy diet is highly recommended to prevent metabolic conditions.

Treatment in adults

Treatment in adults

Most women affected with Turner syndrome need estrogen-progesterone replacement therapy. These treatments aim to provide:

- Normal genital and sexual function with estrogen impregnation
- Good bone health
- Better metabolic health
- Improved overall health and psychological well-being

The treatment can be given as a patch, gel, or tablet.

Weight control, a healthy lifestyle, and regular physical activity are strongly recommended because of the increased risk of obesity and its consequences (metabolic syndrome, high blood pressure, insulin resistance, diabetes).

Any heart defect will be addressed to a specialist, and high blood pressure will be treated.

If associated health disorders such as autoimmune or metabolic disorders have been diagnosed, appropriate treatment will be continued.

Fertility

Fertility

Most women with Turner syndrome cannot have children (are infertile) due to ovarian failure. In patients with spontaneous puberty, ovarian function declines rapidly.

Estrogen-progestogen replacement therapy does not induce ovulation or fertility.

Various options are available to have a child:

- Assisted conception techniques: Oocyte (egg) donation followed by in vitro fertilization is a technique that can be used in many cases. Fertilization is achieved either by fertilizing the egg with sperm (IVF) or by micro-injecting sperm into an egg (ICSI). The embryos are implanted in the mother's uterus.
- Ovarian stimulation followed by oocyte cryopreservation can be proposed in the case of preserved ovarian function.

However, pregnancy may be contraindicated in cases of significant maternal risk (essentially due to severe heart conditions). Pregnancy in patients with Turner syndrome is associated with increased morbidity and mortality in the mother, with the same consequences for the fetus.

Full assessment by cardiologists and obstetricians is indicated.

Adoption might be an option.

Recommended assessment at diagnosis:

Recommended assessment at diagnosis:

Initial assessment	Childhood	Puberty	Adult
Height, weight, BMI, BP, search for strabismus, kyphosis, scoliosis	x	x	x
Skin assessment for nevi	x	x	x
Growth curve (Turner and general population)	x	x	
Puberty and fertility assessment (LH, FSH, E2, AMH, inhibin B, SHBG >depending on ovarian function)	x (only FSH)	x	x
Phosphocalcic and bone health (Calcium, Phosphate, alkaline phosphatase, 25-Vit D, (B-crosslaps P1NP) + bone densitometry		x	x
Liver tests (ASAT, ALAT, γ GT, Bilirubin)	x	x	x
Metabolic profile (HbA1c, fasting blood glucose, fasting insulin)	x (> 10 years)	x	x
Lipids (Chol tot , HDL ; LDL, TG)	x (> 10 years)	x	x
Thyroid function: TSH +/- free T4	> 4 years	x	x
Autoimmune disease research (thyroiditis, celiac disease)	> 4 years	x	x
Neuropsychological assessment	x	x	x
Cardiology consultation	x	x	x
Cardiac ultrasound, Electrocardiogram, Aortic MRI/CT	Cardiac CT/MRI if coarctation or aortic dilatation suspected	Cardiac MRI	x
Renal function and renal US	x	x	x
Pelvic US to look for the ovaries and measure the size of the uterus	x (in the first 6 months of life))	x	x
Thyroid US	x	if dysthyroid or nodular	if dysthyroid or nodular
ENT consultation, Audiogram	x	x	x
US of hips at birth for hip dysplasia	x (at birth)		
Bone age	x	x	
Bone density scan (DEXA)		x	x
Ophthalmological consultation	x /from 12-18 months)	x	x
Dental Consultation	x (from age 7)	x	x
Orthopedic consultation	x (if scoliosis or orthopedic anomaly)	x	
Fertility consultation if ovarian function is preserved		x	x
Fertility consultation if infertile (discuss oocyte donation)			x
Recommend physical activity and healthy diet	x	X	x

Reference: Consensus Turner
Eur J Endocrinol. 2017 Sep;177(3):G1-G70. doi: 10.1530/EJE-17-0430.
Clinical practice guidelines for the care of girls and women with Turner syndrome: proceedings from the 2016 Cincinnati International Turner Syndrome Meeting.

Recommended follow-up examinations

Follow-up examinations

Exam	Childhood	Puberty	Adult
Parameters: height, weight, BMI, BP, kyphosis, scoliosis (from age 8) + Turner growth curve	2x/year	2x/year	1x/year
Bone age	1x/ 1-3 years if on GH	1x/ 1-3 years if on GH	
Growth hormone treatment monitoring (IGF-1, IGFBP3)	1 to 2x/year	1 to 2x/year	
Puberty and fertility assessment (LH, FSH, E2, AMH, inhibin B, SHBG >depending on ovarian function)		1 to 2x/year	
Phosphocalcic and bone balance (Ca, Phosphate, alkaline phosphatase, 25-Vit D, (B-crosslaps P1NP)		1x/year	
Liver tests (ASAT, ALAT, y GT, Bilirubin)		1x/2 year	
Metabolic profile (HbA1c, fasting blood glucose, fasting insulin)	Before treatment with hGH	1x/2 year	
Lipids (total cholesterol, HDL; LDL, TG)	> 10 years : 1x /2 year	1x/2 year	
Thyroid function : TSH +/- free T4	4 years 1y/ year (1x/6 months if hypothyroidism)	1x/year (TSH +/- T4L /6 months if hypothyroidism)	
Autoimmune disease research (thyroiditis, celiac disease)	>4 years: 1x/2 years	1x/2years	
Neuropsychological assessment	1x/year	1x/year	1x/year
Cardiology consultation (depending on cardiac condition)	1x /1-5 years	1x /1-5 years	1x /1-5 year
Cardiac ultrasound, electrocardiogram, aortic MRI (depending on cardiac involvement)	1x /1-5 years	1x /1-5 years	1x /1-5 years
Bone density (DEXA)		Before puberty induction Control at the end of growth or puberty	1x/5 years
Kidney function and renal US	1x	Creatininemia in case of hypertension	
Thyroid US	In case of dysthyroidism, palpation of nodule and/or goiter		
ENT consultation, audiogram	1x	Every 3 years	Every 5 years
Ophthalmological consultation	Around 12-18 months. Control around 2-3 years	According to ophthalmological advice	According to ophthalmological advice
Dental consultation	According to specialized dentist's advice		
Dermatologist	1x	1x year	1x year
Fertility consultation if ovarian function preserved		From age 14, depending on gonadal assessment	1x
Recommend physical activity and healty diet	1x	1x	1x

According to the Turner Consensus reference
Eur J Endocrinol. 2017 Sep;177(3):G1-G70. doi: 10.1530/EJE-17-0430.
Clinical practice guidelines for the care of girls and women with Turner syndrome: proceedings from the 2016 Cincinnati International Turner Syndrome Meeting.

My follow-up

Karyotype:		Date	
Laboratory			
Genetic counseling		Date	
Pre-natal karyotype		Date	

Birth

Birth weight		Birth size	
IUGR : Yes/No			

Cardiological assessment

Problems		NO		YES	Date:	
Detail						
Next check-up					Date:	

Renal evaluation

Problem		NO		YES	Date:	
Detail						
Next check-up					Date:	

Growth

Growth hormone treat-ment		NO		YES	Start date	
Name of treatment					Date end of traitement	
Height (cm)					Date:	
Weight (kg)					Date:	
BMI (Kg/m2)					Date:	
Next check-up					Date:	

Reproductive endocrinology assessment

Spontaneous puberty		NO		YES	Age at onset (years):				
Spontaneous menarche		NO		YES	Age at onset (years):				
Hormone replacement therapy		NO		YES	Age at onset (years):				
Treatment details									
US - pelvic at diagnosis		NO		YES	Ovaries visua-lized		NON		OUI
US - pelvic at onset of spontaneous puberty or induction		NO		YES	Measurement of uterus (cm)				
US - pelvic end of puberty (menarche)		NON		YES					
Pelvic MRI		NON		YES					
Pre-treatment laboratory:		LH		FSH	Date				
		AMH		Inhi-bine B	Date				
Laboratory : last value		LH		FSH	Date				
		AMH		Inhi-bine B	Date				

Orthopedic evaluation

Problem		NO		YES	Date:	
Detail						
Next check-up					Date:	

ENT evaluation / audiogram

Problem		NO		YES	Date:	
Detail						
Next check-up					Date:	

Ophthalmological evaluation

Problem		NO		YES	Date:	
Detail						
Next check-up					Date:	

Orthodontic evaluation

Problem		NO		YES	Date:	
Detail						
Next check-up					Date:	

Lymphedema evaluation

Problem		NO		YES	Date:	
Detail						
Prochain contrôle					Date:	

Psychological evaluation

Problem		NO		YES	Date:	
Detail						
Next check-up					Date:	

Childhood other diagnosis

Thyroiditis		NO		YES	
Coeliac disease		NO		YES	
Thyroid disease other		NO		YES	
Metabolic disease		NO		YES	
Dyslipidemia		NO		YES	
Liver disease		NO		YES	
Bone disease		NO		YES	
Dermatological disease		NO		YES	
Other					

Fertility consultation

		Diagnosed on	
		Date	
		Date	
		Date	

My treatment

Treatment	Dosage	x times a day	Since

My contacts and links

Contacts	Name	City	Phone
Attending physician			
Pediatric endocrinologist			
Adult endocrinologist			
Pediatric cardiologist			
Adult cardiologist			
ENT doctor			
Ophtalmologist			
Psychologist			
Gynecologist			
Fertility specialist			
Other			

Links	
Turner Support Groups - ORPHA	
