# Supplementary material for: Turner syndrome: skin, liver, eyes, dental and ENT evaluation should be improved
Source: Front Endocrinol (Lausanne). 2023 Jul 25;14:1190670. doi: 10.3389/fendo.2023.1190670 (PMC10408677; doi:10.3389/fendo.2023.1190670)
Supplement: Supplementary file 2 [file Table_1.docx]

**Supplementary table 1: Concordance of medical follow-up with the TS health transition passport from the Lausanne University Hospital (CHUV) pediatric and adult endocrine units**

| **Items** | **Monitoring** | **Children** | **Adolescents** | **Adults** | **P-value**^1^ |
| --- | --- | --- | --- | --- | --- |
| Total of patients n=68 |  | 13 | 22 | 33 | NA |
| **Biological n (%)** |  |  |  |  |  |
| Total cholesterol | Each 2 Yr (> 10 Yr of age) | 100^2^ | 81.8 | 81.8 | 1 |
| HDL, LDL, TG | Each 2 Yr (> 10 Yr of age) | 100^2^ | 77.3 | 81.8 | 0.790 |
| Créatinine, Urea | At least once for children, if high blood pressure for adolescents and adults | 69.2 | 92.3^3^ | 89.3^3^ | 0.369 |
| IGF-BP3 concentration (on GH treatment) | Annually (< 18 Yr of age) | 70^4^ | 83.3^4^ | NA | 1 |
| Oestradiol | Annually for adolescents | NA | 45.5 | NA | NA |
| LH, FSH | Annually for adolescents | NA | 77.3 | NA | NA |
| AMH | Annually for adolescents | NA | 36.4 | NA | NA |
| Inhibin B | Annually for adolescents | NA | 22.7 | NA | NA |
| **Radiological n (%)** | | | | | |
| Thyroid US | At least once if dysthyroidism | NA^5^ | 0^6^ | 38.5^6^ | 1 |
| Bone age (on GH treatment) | Each 1-3 Yr (< 18 Yr of age) | 80^4^ | 94.4^4^ | NA | 1 |

^1^The p-values were calculated across groups: “Children”, “Adolescents” and “Adults” with the Fisher’s Exact Test, ^2^Only one girl > 10 Yr of age,  ^3^13 adolescents and 28 adults with high blood pressure, ^4^10 children and 18 adolescents under GH treatment, ^5^No patient with dysthyroidism so no thyroid US done, ^6^1 adolescents and 13 adults with dysthyroidism

Abbreviations: AMH: Anti-Müllarian hormone; FSH: Follicle Stimulating Hormone; HDL: High Density Lipoproteins; IGF-BP3: Insulin-like Growth Factor-Binding Protein 3; LDL: Low Density Lipoproteins; LH: Luteinizing Hormone; TG: Triglyceride; TTE: Transthoracic echocardiography: US: Ultrasound
